# Bromide of Potassium in Epilepsy

**Published:** 1869-07

**Authors:** William A. Hammond

**Affiliations:** New York


### Bromide of Potassium in Epilepsy.

BY WILLIAM A. HAMMOND, M. D., NEW YORK.

In regard to the dose of bromide of potassium in epilepsy, Dr. Hammond states that the symptoms due to large doses of the bromide may be enumerated as follows, in the usual order of their occurrence: 1. Contraction of the pupils; 2. Drowsiness; 3. Weakness of the arms and legs; 4. Depression of mind; 5. Failure of memory; 6. Delusions. The first three of these are the usual accompaniments of a dose of the medicine capable of producing any influence over epilepsy. In adults they never follow less doses than ten grains. Doses of five grains produce no effect.

*Hospital of Diseases of the Chest, London.*

| | | | |
|---|---|---|---|
| 132. | ℞. | Potassii bromidi, . . . . . | gr. x. |
| | | Tincture conii, . . . . . . | ♏ xxx. |
| | | Tincture valerianæ ammoniatæ, . | ♏ x. |
| | | Aquæ camphoræ, . . . . . . | f ℥ j. |

For one dose, ter die.

*Hospital of University College, London.*

| | | | |
|---|---|---|---|
| 133. | ℞. | Potassi bromidi, . . . . . | gr. x. |
| | | Spirits chlorformi, . . . . . | ♏ xviij. |
| | | Infusi quassiæ, . . . . . . | f ℥ j. ♏. |

For one dose, ter die.

*Dr. Marshall Hall.*

| | | | |
|---|---|---|---|
| 134. | ℞. | Strychniæ acetatis, . . . . . | gr. j. |
| | | Acidi acetic, . . . . . . . | ♏ xx. |
| | | Alcholis, . . . . . . . . | f ʒ ij. |
| | | Aquæ distillatæ, . . . . . . | f ʒ vi. ♏. |

Ten drops (= gr. 1-50) to be taken in water ter die.

